# When Depression Mimics Dementia: A Case of Acute Cognitive Decline in Later Life

**DOI:** 10.1192/bjo.2026.11788

**Published:** 2026-06-30

**Authors:** Maneesha Kallukkadan Paul

**Affiliations:** LSCFT, Blackburn, United Kingdom

## Abstract

**Aims:**

Rapid cognitive decline in later life often prompts urgent assessment for neurodegenerative dementia. However, severe depression and anxiety can produce profound but potentially reversible cognitive impairment, frequently described as depressive pseudodementia. Presentations characterised by abrupt onset, marked functional impairment, prominent subjective memory complaints, preserved insight, and disproportionate distress should raise suspicion for affective pathology. Failure to recognise reversible causes can lead to misdiagnosis, unnecessary investigations, and distress for patients and families.

**Methods:**

Mr X is a 71-year-old White British retired man who presented with a two-week history of rapidly progressive cognitive and functional decline. He reported severe memory impairment, including inability to recall his date of birth or home address. He became unable to perform basic activities of daily living, forgetting how to make a cup of tea, operate household appliances, manage finances or shop independently. He frequently lost track of tasks midway and described episodes of intending to shower but later realising he had not done so.

He had recently commenced tirzepatide (Mounjaro) for weight loss, raising family concern about potential cognitive effects. Six-CIT score was 24/28. Blood investigations, including thyroid function, vitamin levels, and routine metabolic screening, as well as CT brain imaging, were unremarkable. He was referred to memory services for assessment.

During further reviews with the secondary care team, it was evident that affective symptoms were prominent. He was intensely anxious, preoccupied with fears of dementia, and distressed about becoming dependent or forgetting his family. He described low mood, anhedonia, early morning waking, appetite disturbance, and longstanding passive suicidal ideation. DASS-21 scores indicated severe depression, anxiety, and stress. Cognitive symptoms fluctuated in parallel with anxiety. Treatment with sertraline, alongside biopsychosocial support, led to early improvement in mood, confidence, and cognitive clarity.

**Results:**

The abrupt onset, fluctuating deficits, preserved insight, and overwhelming affective distress were inconsistent with neurodegenerative dementia. Anxiety-driven hypervigilance likely perpetuated impairment. Memory assessments conducted during severe mood and anxiety disturbance may be misleading. Treating underlying mood and anxiety through pharmacological and non-pharmacological interventions can substantially improve cognitive function.

**Conclusion:**

This case highlights the importance of recognising late-life depression and anxiety as reversible mimics of dementia. Clinicians should routinely screen for reversible causes of cognitive impairment, including depression, nutritional deficiencies, hypothyroidism, medication effects, infection, alcohol misuse, and electrolyte disturbances, to avoid misdiagnosis and support recovery.

